# Resveratrol counteracts lipopolysaccharide-induced depressive-like behaviors *via* enhanced hippocampal neurogenesis

**DOI:** 10.18632/oncotarget.11178

**Published:** 2016-08-10

**Authors:** Liang Liu, Qin Zhang, Yulong Cai, Dayu Sun, Xie He, Lian Wang, Dan Yu, Xin Li, Xiaoyi Xiong, Haiwei Xu, Qingwu Yang, Xiaotang Fan

**Affiliations:** ^1^ Department of Developmental Neuropsychology, School of Psychology, Third Military Medical University, Chongqing, China; ^2^ Department of Neurology, Xinqiao Hospital, Third Military Medical University, Chongqing, China; ^3^ Southwest Eye Hospital, Southwest Hospital, Third Military Medical University, Chongqing, PR China

**Keywords:** resveratrol, depression, neurogenesis, LPS, radial glial cells, Pathology Section

## Abstract

Radial glial-like cells (RGLs) in the adult dentate gyrus (DG) function as progenitor cells for adult hippocampal neurogenesis, a process involved in the stress-related pathophysiology and treatment efficiency of depression. Resveratrol (RSV) has been demonstrated to be a potent activator of neurogenesis. The present study investigated whether chronic RSV treatment has antidepressant potential in relation to hippocampal neurogenesis. Mice received two weeks of RSV (20 mg/kg) or dimethylsulfoxide (DMSO) treatment, followed by lipopolysaccharide (LPS; 1 mg/kg) or saline injections for 5 days. We found that RSV treatment abrogated the increased immobility in the forced swimming test and tail suspension test induced by LPS. Immunohistochemical staining revealed that RSV treatment reversed the increase in microglial activation and the inhibition in DG neurogenesis. RSV treatment also attenuated LPS-induced defects in the expanding of RGLs through promoting symmetric division. In addition, RSV ameliorated LPS-induced NF-κB activation in the hippocampus coincides with the up-regulation levels of Sirt1 and Hes1. Taken together, these data indicated that RSV-induced Sirt1 activation counteracts LPS-induced depression-like behaviors *via* a neurogenic mechanism. A new model to understand the role of RSV in treating depression may result from these findings.

## INTRODUCTION

Depression is a chronic, recurring, and life-threatening mood disorder, which affects more than 14 percent of the population and imposes a tremendous burden on both individuals and society [1]. Clinically, depression is characterized by anhedonia, sleep disturbances, reduced food intake, decreased locomotor activity, and increased sensitivity to pain [2]. The etiology of depression has not been fully elucidated and the biological mechanisms remain unclear [3].

Adult hippocampal neurogenesis in the subgranular zone (SGZ) of the DG contributes to mood regulation [4]. Brain imaging and postmortem studies of patients suggest that reduced DG size may be related to decreased neurogenesis and mature neuronal cell loss [5]. Impaired hippocampal neurogenesis also occurs in some rodent and non-human primate models of depression such as repeat restraint stress, chronic unpredictable mild stress, social defeat stress, social isolation, and LPS or corticosterone administration [6-8]. A recent study by Fava et al. found that a novel neurogenic compound, NSI-189, effectively treated major depressive disorder [9]. Thus, these findings indicated that reduced adult hippocampal neurogenesis may be involved in the pathological mechanisms of depression and that up-regulating neurogenesis is a potential therapeutic target for treating depression.

The neural stem cells (NSCs) in the SGZ are classified into types 1 and 2. Type 1 cells have a radial glia-like (RGL) morphology and express both glial fibrillary acidic protein (GFAP) and SOX2; type 2 cells express only SOX2, and can function as the precursor cells to neurons or astrocytes [10, 11]. Several lines of evidence have demonstrated that the GFAP-expressing RGLs determine the type of NSCs in postnatal and adult brain, and provide the principal source of constitutive neurogenesis in the adult hippocampus by increasing their capability for proliferation and/or survival in response to stimuli and environmental demands [12, 13]. However, the mechanisms to maintain the neurogenesis capacity of these RGLs in response to stress have not been fully elucidated.

Resveratrol (RSV) is a stilbene compound that is found in the skin of red grapes and in certain medicinal plants [14, 15]. Recently, RSV was reported to promote the survival of adult hippocampal NSCs in a mouse model of neuroinflammation [16]. Similarly, our previous study confirmed that RSV activation of Sirtuin type 1 (Sirt1) blocked the decline of hippocampal neurogenesis induced by ethanol exposure during early postnatal life [17]. Indeed, RSV alone increased proliferation of RGLs in the postnatal SGZ [18]. Moreover, recent studies have found that RSV ameliorated depressive-like behaviors in animal models [19-21]. The role of RSV in promoting hippocampal neurogenesis, including potential anti-depressant-like properties, has not been investigated.

In the present study, an LPS induced stress-based depressive mouse model [6, 22, 23] was used to test the antidepressant effects of RSV. Our findings revealed that pretreatment with RSV for 2 weeks reversed LPS-induced depression-like behaviors. The antidepressant effects of RSV coincide with reduced LPS-induced inflammation and inhibition of hippocampal neurogenesis. Furthermore, we present the first demonstration that RSV activation of Sirt1 reverses the LPS-induced reductions in the number of RGL progenitor cells in the SGZ by stimulating symmetric division. Therefore, our results indicate a novel mechanism for RSV regulation of adult neurogenesis and consequent depressive-like behavior induced by LPS.

## RESULTS

### RSV alleviates LPS-induced depression-like behaviors

**Figure 1:**
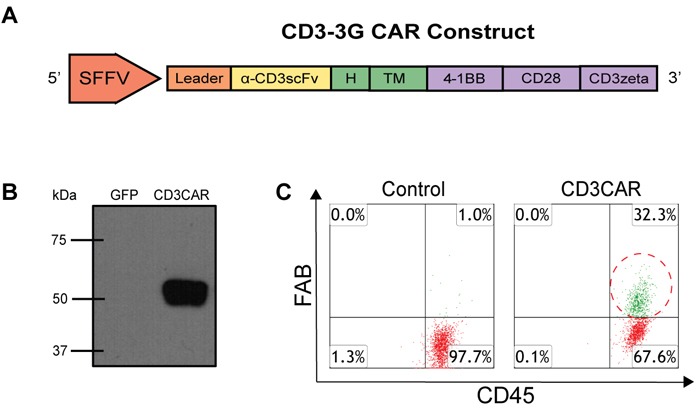
Effects of RSV on the duration of immobility on the forced swimming test (FST) and tail suspension test (TST) in LPS injected mice **A.** and **B.** LPS induced depression-like behaviors on the FST and TST. **A.** LPS treated mice displayed more immobility time on the FST, and pretreatment with RSV reversed this effect (*n* = 8). **B.** LPS treated mice displayed an increased duration of immobility on the TST, and pretreatment with RSV reversed this effect (*n* = 8). Data are presented as mean ± SEM.*p < 0.05; **p < 0.01.

The TST and FST tests were widely used to evaluate depression-like behaviors in mice [24, 25], and increased immobility in both the TST and FST is a measure of behavioral despair. As shown in Figure 1A, there were significant differences between the 4 groups for the duration of immobility during the FST (*F_3, 28_* = 38.678, *p* < 0.01). Post hoc analyses indicated that LPS injected animals (LPS+DMSO) demonstrated significantly more immobility during the FST compared to saline treated control mice (Saline+DMSO) (*p* < 0.01). Pretreatment with RSV (LPS+RSV) significantly reduced the immobility period compared to the LPS treated group (*p <* 0.01), however, RSV did not alter immobility durations in the control animals.

In order to confirm the FST results, we analyzed immobility duration using the TST (Figure 1B) and found that LPS injected mice were immobile for a significantly longer duration of time than saline treated control mice (*p* < 0.01). Pretreatment with RSV markedly reduced the duration of immobility on the TST in LPS injected mice (*p* < 0.05).

**Figure 2:**
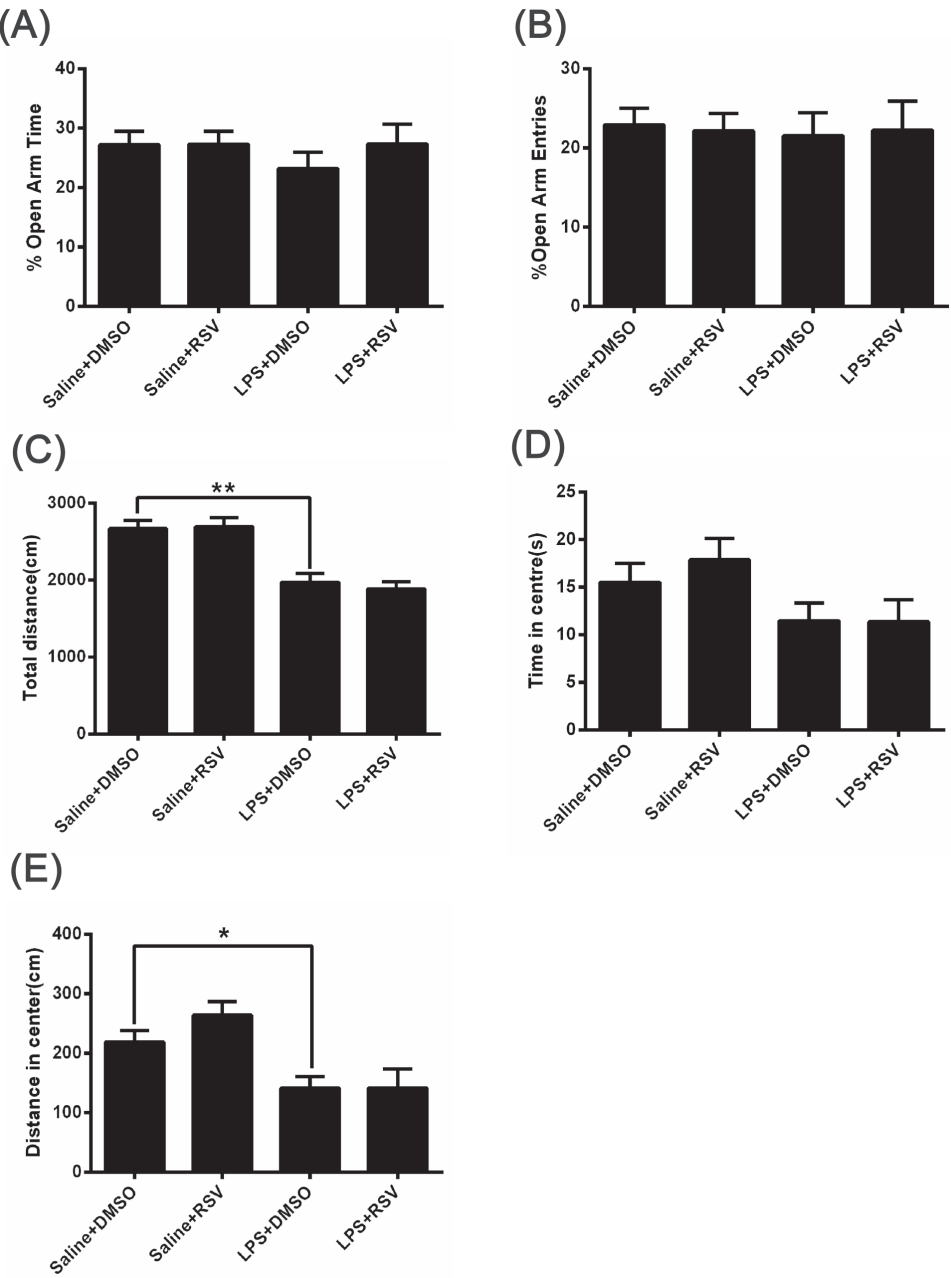
The effect of RSV on LPS injected mice in the elevated plus-maze test and the open field test **A.** and **B.** Behavior of the 4 groups of mice in the elevated plus-maze test. There were no difference in the percentage of time spent in the open arms (A) or the percentage of open arm entries (B) between the 4 groups (*n* = 8). **C.**-**E.** Behavior of the 4 groups of mice in the open field test. LPS treatment decreased the overall distance traveled (C) and distance in center (E), but there were no significant differences in the duration of time spent in the center of the field (D). Data are presented as mean ± SEM.*p < 0.05; **p < 0.01.

We next examined a cohort of mice using the elevated plus maze, a common test of anxiety-like behavior in rodents [26]. There were no significant differences in the percentage of time spent in open-arms (*F_3, 28_* = 2.512, *p* > 0.05; Figure 2A) or percentage of open arm entries (*F_3, 28_* = .042, *p* > 0.05; Figure 2B) between the 4 groups. On the open field test, there were no significant differences between the 4 groups for the duration of time spent in the center of the field (*F_3, 28_* = 2.287, *p* > 0.05; Figure 2D). However, as shown in Figure 2C, there were significant differences for total distance [*F*_3, 28_ = 16.173, *p* < 0.01]. LPS treatment significantly decreased the total distance (*p* < 0.01; Figure 2C) and distance in center (*p* < 0.05; Figure 2E), but pretreatment with RSV did not block these effects (*p* > 0.05).

### RSV inhibits LPS-induced over-activation of microglia in the DG-SGZ

**Figure 3:**
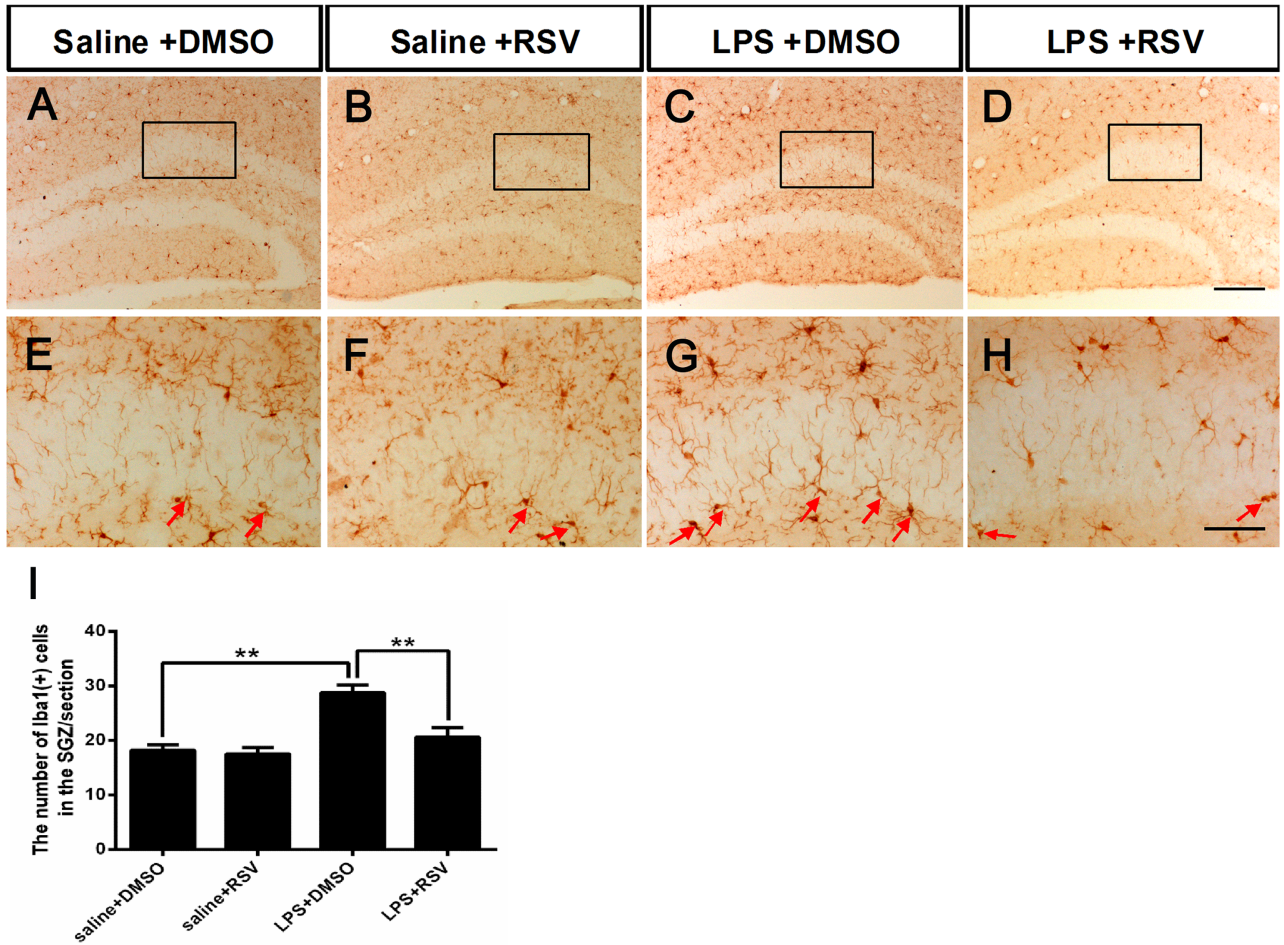
Pretreatment with RSV inhibited LPS-induced over-activation of microglia in the SGZ **A.**-**D.** Microglia in the SGZ of the 4 groups, as shown by immunostaining with the microglia-specific marker Iba1. **E.**-**H.** Higher-power views of the boxed areas in (A-D). Arrowheads indicate Iba1^+^ cells in the SGZ. **I.** The number of Iba1^+^ cells in the SGZ. Data are presented as mean ± SEM (*n* = 5). **p < 0.01. Scale bar in D = 200 μm and applies to (A-D), in H = 20 μm and applies to (E-H).

Microglial activation in the SGZ was assessed using ionized calcium binding adapter molecule 1 (Iba-1) staining, which demonstrated that the number of Iba-1 immunoreactive cells in the DG-SGZ increased significantly following LPS injection, compared to all other groups (*F*_3,16_ = 14.714, *p* < 0.01; Figure 3). Iba-1 positive microglia in the hippocampus of saline-treated control animals had smaller cell bodies with fewer and scattered processes (Figure 3A, 3E), but LPS injected mice had enlarged cell bodies with thicker and more condensed processes (Figure 3C, 3G). RSV pretreatment resulted in reduced microglia with activated morphologies following LPS injection (Figure 3D, 3H). RSV alone did not alter Iba-1 positive microglia in the hippocampus of the intact animals (Figure 3B, 3F). Analyses of Iba-1 staining data confirmed that LPS injection increased Iba-1 immunoreactivity in the SGZ and that RSV pretreatment had an inhibitory effect (Figure 3I).

### RSV prevented LPS-induced suppression of hippocampal neurogenesis

**Figure 4:**
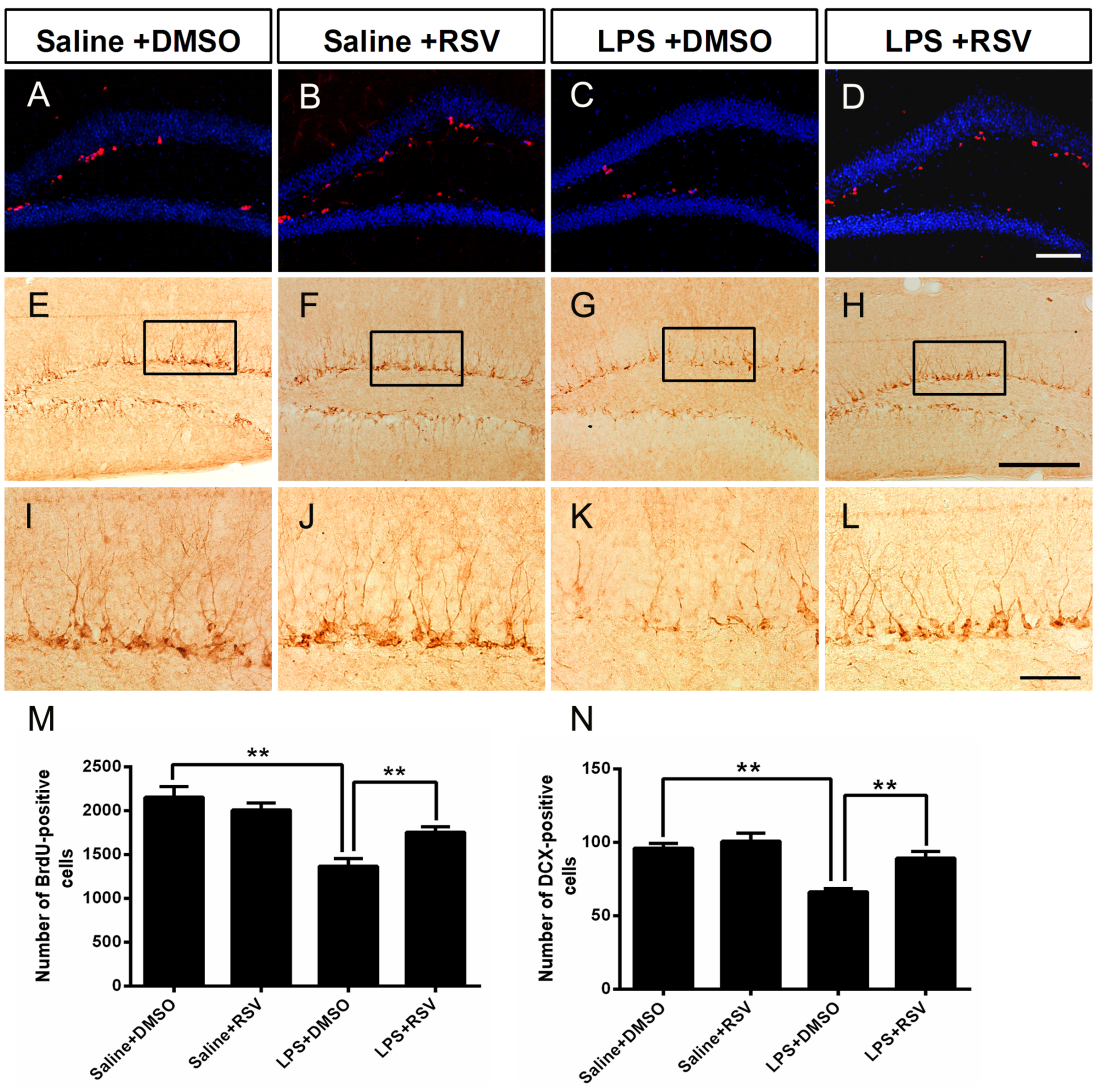
Pretreatment with RSV prevented the LPS-induced decrease of BrdU-positive cells and DCX-positive cells in the SGZ **A.**-**D.** BrdU-positive cells in the SGZ for each of the 4 groups. Mice were sacrificed 24 h after the last BrdU injection. **E.**-**H.** Doublecortin positive (DCX^+^) cells in the SGZ. **I.**-**L.** Higher-power views of the boxed areas in E-H. RSV treatment reversed the LPS-induced decrease of BrdU-positive cells **M.** and DCX-positive cells **N.** Data are presented as mean ± SEM (*n* = 5). **p < 0.01. Scale bar in (D) = 100 μm and applies to (A-D), in (H) = 200 μm applies to (E-H) and in (L) =20μm applies to (I-L).

Reduced adult DG neurogenesis contributes to depression-like behaviors [27], while increased hippocampus neurogenesis alleviates depression-like behaviors [28].Therefore, we analyzed the proliferating cell population in the SGZ using the BrdU incorporation assay, which demonstrated that there was a significant treatment effect for the number of BrdU^+^ cells (*F_3, 16_* = 5.29, *p* <0.05; Figure 4A-4D and 4M). Post hoc analyses indicated that LPS treatment significantly decreased the number of BrdU^+^ cells in the SGZ compared to the saline treated control group (*p* < 0.01), while RSV increased the number of BrdU^+^ cells in the LPS treated group (*p* < 0.01), but not in the control group (*p* > 0.05). New granular neurons are produced continuously, and originate in the SGZ of the DG. DCX immunoreactivity is a method to identify immature neurons, therefore, we assessed DCX positive immature neurons in the SGZ. We found a significant treatment effect for the number of DCX positive immature neurons in the SGZ (*F_3, 16_* = 14.323, *p* < 0.01; Figure 4E-4L and 4N), with LPS treated mice possessing significantly fewer DCX positive cells than mice in the saline treated control group (*p* < 0.01). However, pretreatment with RSV reversed this effect (*p* < 0.01).

**Figure 5:**
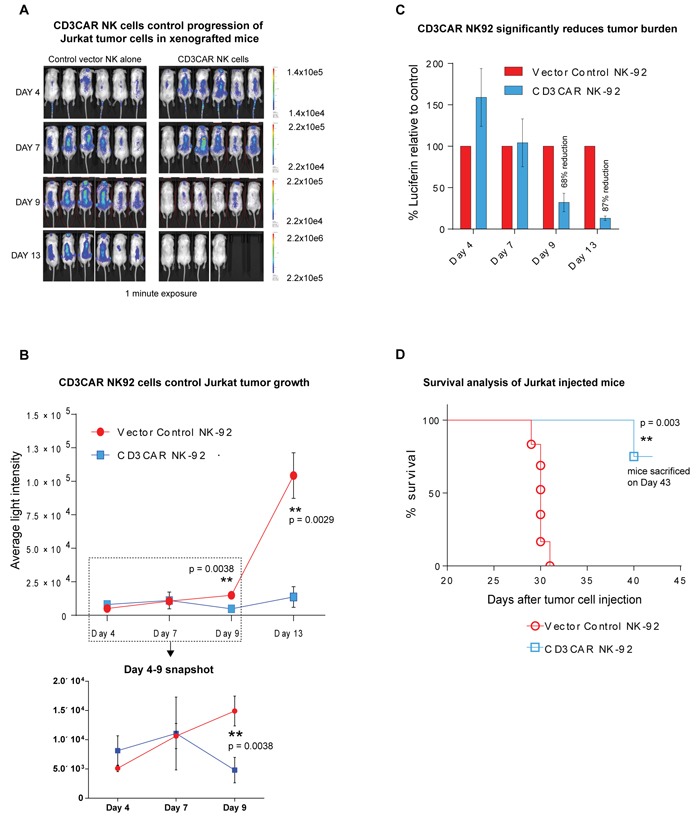
Pretreatment with RSV prevented the decrease of BrdU-positive cells, as well as the number of cells positive for both BrdU and DCX in the DG at 14 d following BrdU injection **A.**-**D.** BrdU-positive cells and DCX-positive cells in the DG. Mice were sacrificed 14 d after the last BrdU injection. RSV treatment reversed the decrease of BrdU-positive cells **E.**, as well as BrdU and DCX double-positive cells **F.** induced 14 d following LPS injection. Data are presented as mean ± SEM (*n* = 5).*p < 0.05. Scale bar in (D) = 20 μm and applies to (A-D).

We further analyzed the number of BrdU^+^ nuclei, as well as cells positive for both BrdU and DCX, in the DG at 14 d following BrdU injections (Figure 5). Exposure to LPS significantly decreased the number of BrdU^+^ cells (*p* < 0.05), and this effect was completely blocked by RSV (*p* < 0.05) (Figure 5E). Analysis of the BrdU^+^/DCX^+^ double-labeled cells demonstrated that LPS also decreases the number of immature neurons (*p* < 0.05), and this effect was blocked by RSV (*p* < 0.05) (Figure 5F). These data indicate that RSV treatment prevented LPS-induced impairment of hippocampal neurogenesis.

### RSV alleviates LPS-mediated exhaustion of NPCs in the DG-SGZ

**Figure 6:**
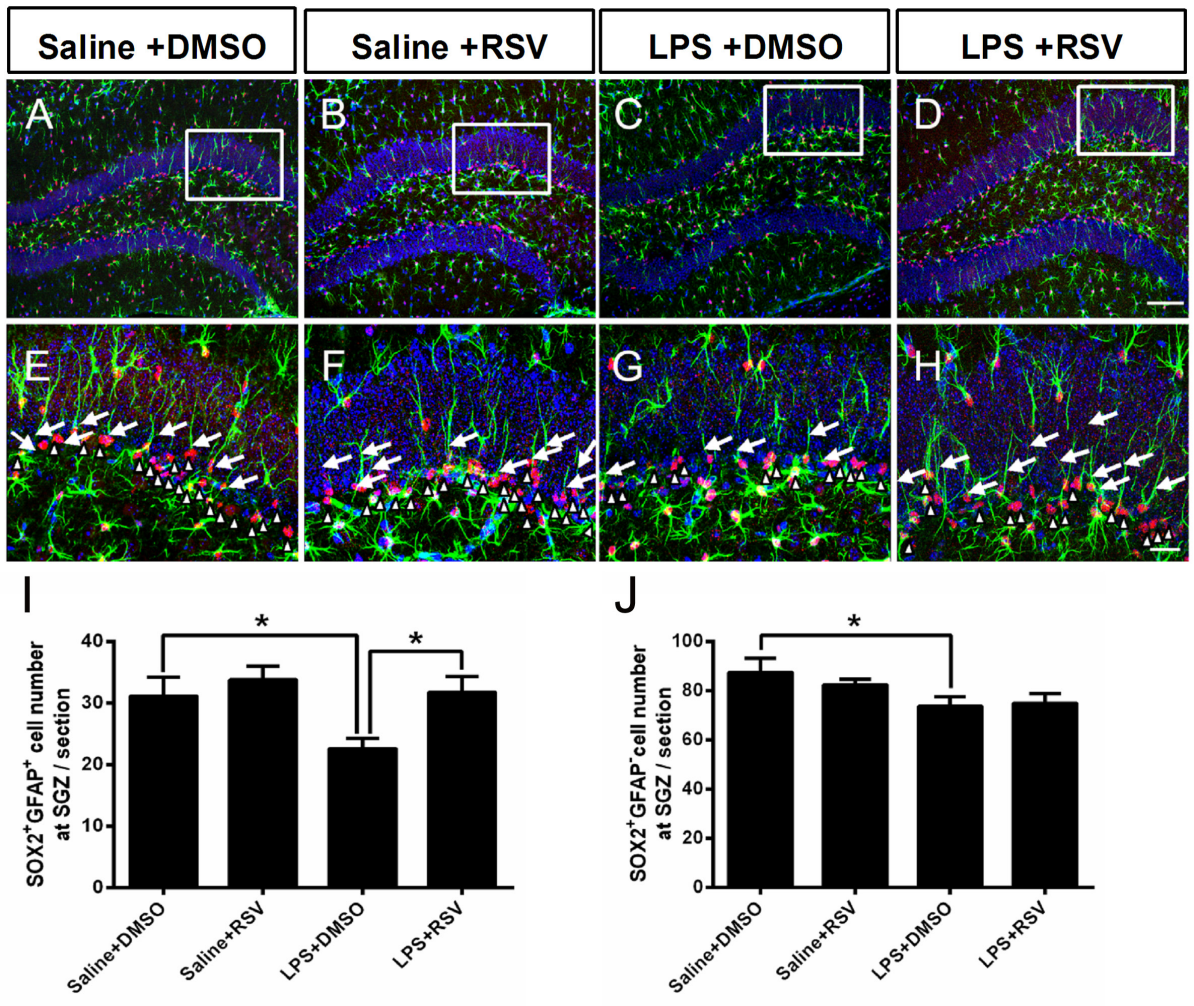
Pretreatment with RSV inhibited the LPS-induced decrease in neuronal stem cells in the SGZ **A.**-**D.** SOX2 and GFAP double immunostaining in the DG-SGZ for each of the 4 groups. **E.**-**H.** Higher-power views of the boxed areas in (A-D). Arrowheads indicate SOX2^+^GFAP^+^ cells in the SGZ. White triangle decorators indicate SOX2^+^GFAP^-^ cells in the SGZ. LPS treatment decreased the number of SOX2-positive and GFAP-positive (SOX2^+^GFAP^+^) cells **I.**, as well as SOX2-positive and GFAP-negative (SOX2^+^GFAP^-^) cells **J.** in the SGZ, but pretreatment with RSV only inhibited the LPS-induced (SOX2^+^GFAP^+^) cell decrease. Data are presented as mean ± SEM (*n* = 5).*p < 0.05. Scale bar in D = 100 μm and applies to (A-D), in H = 25 μm and applies to (E-H).

In the adult hippocampus, a reduction in cellular proliferation in the SGZ of the DG may result from a decline in available NSCs. NSCs primarily include 2 types of NSCs: SOX2 and GFAP double-positive (SOX2^+^/GFAP^+^) cells (type 1), and SOX2-positive and GFAP-negative (SOX2^+^/GFAP^−^) cells (type 2) [10, 11]. In order to explore the treatment effects on NPCs, we used SOX2 and GFAP double-labeling, with two-way ANOVA demonstrating significant differences between the 4 groups (*F_3, 16_* = 4.203, *p* < 0.05; Figure 6I). Post-hoc analyses indicated that LPS treatment significantly decreased the number of (SOX2^+^/GFAP^+^) cells in the SGZ compared to the saline treated control group (*p* < 0.05), whereas RSV increased the number of SOX2^+^/GFAP^+^cells in LPS treated group (*p* < 0.05), but not in the control group (*p* > 0.05). There were no significant differences between groups for SOX2^+^/GFAP^−^ cells in the SGZ (*F_3, 16_* = 2.421, *p* = 0.104; Figure 6J). Specifically, LPS treatment decreased SOX2^+^/GFAP^−^ cells in the SGZ (*p* <0.05), but this effect was not reversed by RSV pretreatment (*p* > 0.05). RSV primarily reversed LPS-induced reductions of type 1 NSCs in the DG, indicating that maintenance of the NSC population impacts RGLs.

### RSV modulates LPS-induced imbalances between symmetric and asymmetric division patterns of RGL progenitor cells in the DG-SGZ

**Figure 7:**
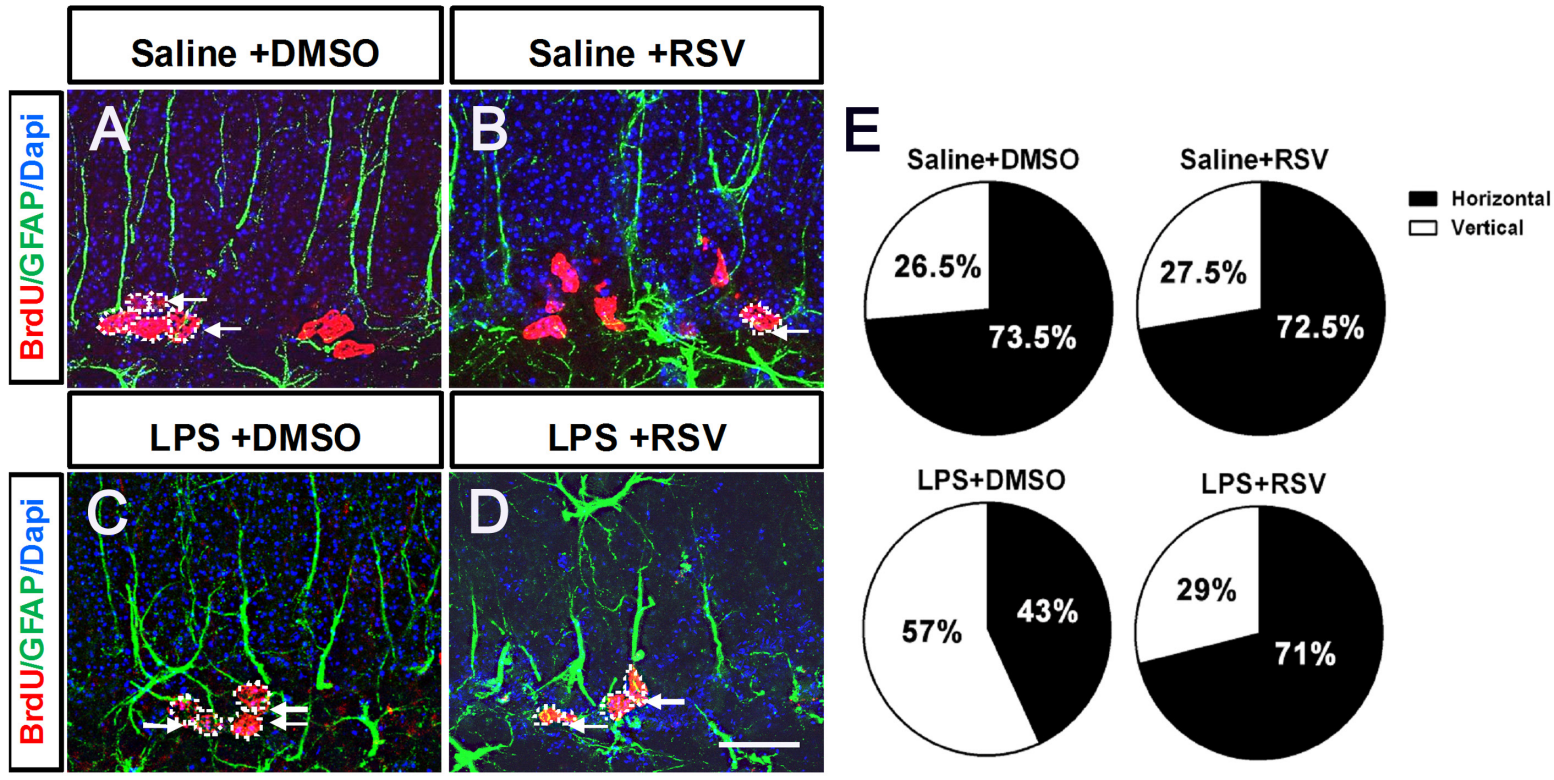
Pretreatment with RSV modulated the division mode of RGL progenitor cells in the GCL that was altered by LPS **A.**-**D.** BrdU and GFAP double immunostaining in the GCL for each of the 4 groups. There were differently aligned patterns of BrdU-labeled RGL progenitor cells, including horizontally and vertically aligned patterns. Single arrowhead indicates horizontally aligned patterns. Two arrowheads arranged along the up-down direction indicate vertically aligned patterns. **E.** Quantitative analysis of the cell alignment modes between the 4 groups. LPS treatment decreased the horizontally aligned pattern compared to the other groups (Saline+DMSO, 77.5%; LPS+DMSO, 39.5%. *p* < 0.01), and pretreatment with RSV reversed this effect (LPS+DMSO, 39.5%; LPS+RSV, 76%. *p* < 0.01). Data are presented as mean ± SEM (*n* = 5). Scale bar in D = 20 μm and applies to (A-D).

Type 1 RGLs in the DG can self-renew in adults by either asymmetric or symmetric cell division, therefore, NSCs are maintained throughout adulthood [11]. The modes of cell division of RGLs are essential to hippocampal neurogenesis. Two examples of these modes are asymmetric cell division, which results in a single daughter neuron and a mother cell that remains a progenitor cell, and symmetric cell division, which increases the number of progenitor cells [29, 30]. In order to investigate the effects of RSV and LPS on the mode of cell division, we examined alignment of the newly generated pairs of RGL progenitor cells in the granule cell layer (GCL) 1 d after BrdU injections. We found that the LPS treatment group had a significantly lower percentage of symmetric division compared to the control group (Saline+DMSO, 77.5%; LPS+DMSO, 39.5%; *p* < 0.01; Figure 7E). Furthermore, pretreatment with RSV alleviated the effects induced by LPS (LPS+DMSO, 39.5%; LPS+RSV, 76%. *P* < 0.01; Figure 7E), which indicates that RSV may alter the RGL progenitor cells division mode, thereby influencing the available progenitor cells.

### RSV reverses LPS-induced increases in NF-κB and decreases Hes1 and Sirt1 protein expression in the hippocampus

**Figure 8:**
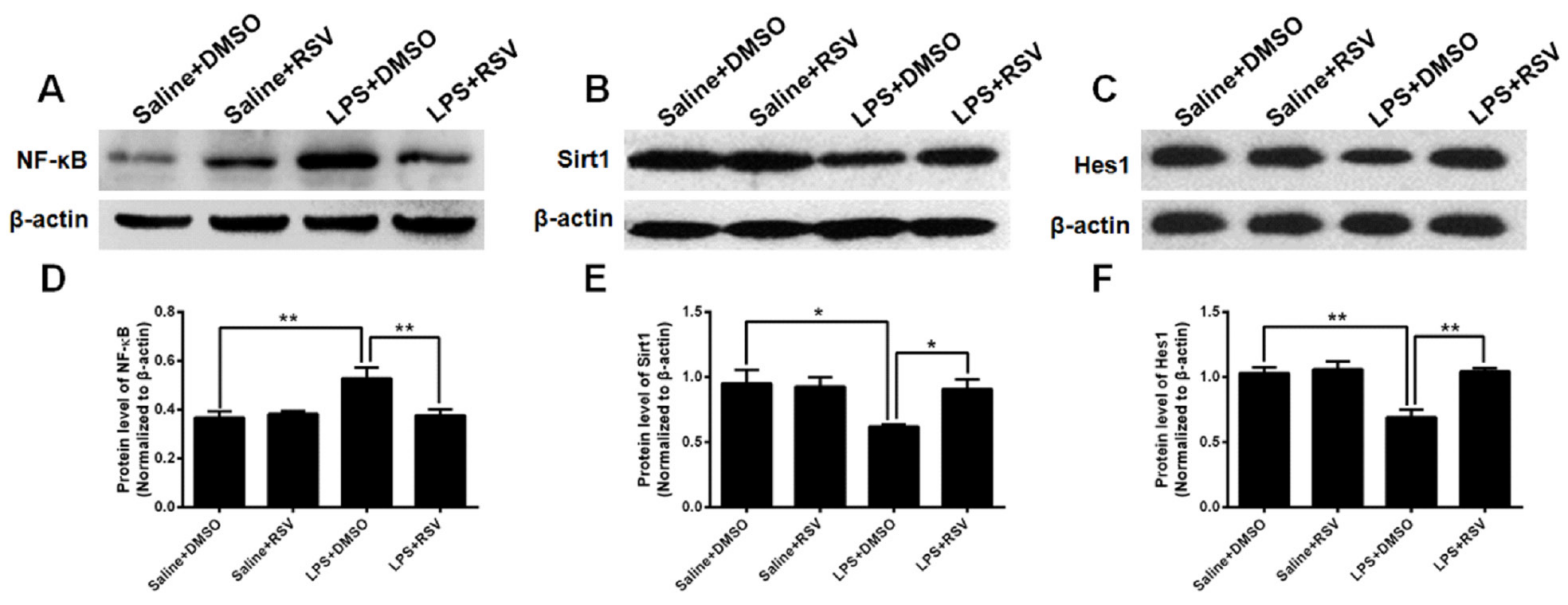
Expression of NF-κB, Sirt1, and Hes1 in the mouse hippocampus **A.** and **B.** Western blots assessing NF-κB and Sirt1 expression in the hippocampus of the 4 groups. LPS treatment significantly increased NF-κB expression in the hippocampus, whereas pretreatment with RSV inhibited this effect **D.** Conversely, compared to the saline treated group, LPS treatment decreased Sirt1 expression in the hippocampus, whereas pretreatment with RSV reversed this effect **E.** **C.** Western blots assessing Hes1 expression in the hippocampus of the 4 groups. Hes1 was significantly decreased in the hippocampus of LPS treated mice, whereas pretreatment with RSV prevented this effect **F.** Data are presented as mean ± SEM (*n* = 3).*p < 0.05; **p < 0.01.

The Sirt1/NF-κB signaling pathway is involved in LPS-induced inflammation [31], therefore, we performed western blotting to investigate whether RSV treatment inhibits LPS-induced inflammation via this signaling pathway. Hippocampal protein expression of NF-κB was significantly different between the 4 groups (*F_3, 8_* = 6.868, *p* < 0.05; Figure 8A, 8D), and post-hoc analyses demonstrated that NF-κB protein expression was up-regulated in the LPS treatment group compared to the saline treated control group (*p* < 0.01). Furthermore, pretreatment with RSV repressed NF-κB protein expression in the hippocampus (*p* < 0.01). Therefore, there were marked between-group differences for Sirt1 protein expression in the hippocampus (*F*_3, 8_ = 4.462, *p* < 0.05; Figure 8B, 8E). Compared to saline treated control mice, Sirt1 protein expression was significantly decreased (*p* < 0.05), but pretreatment with RSV reversed this effect (*p* < 0.05).

The Hairy and Enhancer of Split homologue-1 (Hes1) is essential to the maintenance of RGLs [32], so we also assessed Hes1 protein expression in the mice hippocampus, with findings demonstrating differences between the 4 groups (*F_3, 8_* = 13.094, *p* < 0.01; Figure 8C, 8F). Specifically, LPS treatment significantly decreased Hes1 protein expression compared to the saline treated control group (*p* < 0.01), but pretreatment with RSV reversed the effect (*p* < 0.01). In conjunction, the results indicate that RSV reverses LPS-induced inhibition of Hes1 expression. Furthermore, this effect may occur via regulation of the Sirt1/NF-κB signaling pathway.

## DISCUSSION

In the present study, we found that LPS treatment increased depressive-like behaviors, as assessed by the TST and FST, without affecting anxiety-like behaviors. The findings also corroborate previous reports demonstrating that RSV ameliorates depression-like behaviors in mice, which were induced by repeated corticosterone [19] and LPS [21]. The anti-depressant activities of RSV in the present experiments do not appear to be the result of an overall attenuation of psychomotor activity, as locomotor activity was unaffected by RSV treatment.

It should be noted that behavioral changes did not occur in naïve mice, rather, RSV attenuated behavioral changes induced by i.p. LPS. We observed RSV pretreatment also decreased Iba-1 labeling of microglia in the SGZ elicited by LPS, which may be partially responsible for the observed anti-depressant activity. We also demonstrated a decrease in the number of proliferating SGZ cells following LPS administration. Previous research has indicated that LPS-induced neuroinflammation reduces neurogenesis in the hippocampus [33, 34]. Gebara et al. found an inverse correlation between the number of microglia present in the hippocampus and NSC proliferation [35]. Consistent with these findings, we have previously demonstrated that memantine promotes hippocampal neurogenesis in APP/PS1 mice, partially by inhibiting microglial over-activation [36]. It has been reported that RSV protected neurons by suppressing microglial activation [37]. Unexpectedly, RSV effectively reversed suppression of hippocampal neurogenesis following LPS. Similarly, Kodali et al have confirmed that RSV-treated animals displayed increased net and diminished microglial activation in the hippocampus [38]. Meanwhile, we also noticed that RSV had no effect in animals not exposed to LPS. There are discrepancies in the effect of RSV on DG neurogenesis of intact mice. Torres-Pérez et al has found 6 months of age female Balb/C mice received two weeks of RSV (40 mg/kg) increased DG neurogenesis [39]. However, another study from Park et al have demonstrated that male C57BL/6 mice (4 weeks old) received two weeks of RSV (1-10 mg/kg) inhibited DG neurogenesis [40]. Probably, the different dosage of RSV and mice of age, sex and genetic background could produce different effects on intact animals.

The available NSCs determine the capacity of adult hippocampal neurogenesis, and we found that LPS treatment decreased both types of NSCs in the SGZ, but that pretreatment with RSV only reversed the effect in type 1 cells. Whether type 1 cells convert to type 2 cells is not fully understood [10, 11], it is very likely that RSV rescued the deficits of the self-renewal capacity of type 1 cells induced by LPS which may contribute to the recovered neurogenesis in LPS-injected mice.

RGLs in the SGZ experience 2 types of division patterns: asymmetrical and symmetrical patterns. Asymmetrical division patterns produce differentiation of RGLs into astrocytes or neurons, thus exhausting the available stem cells in the DG-SGZ of the hippocampus, whereas symmetrical division increases the number of RGL progenitor cells and enhances neurogenesis [29, 30]. In the present study, RSV increased the population of RGL progenitor cells by promoting symmetrical division. These findings indicate that RSV reversed the LPS-induced decrease in hippocampal neurogenesis by initiating continuous neurogenesis via symmetrical division. Other contributors to enhanced neurogenesis such as antidepressants, running, and enriched environments may not increase the number of the primary progenitor cells [41-43].

Although changes to division modes of RGLs have been observed under various conditions, the molecular mechanisms controlling the balance of symmetric and asymmetric division had not been fully elucidated. The Notch1/Hes1 signaling pathway has been implicated in regulation of RGL division patterns [44, 45]. The absence of the Hes1 gene leads to decreased NPCs in the DG, a reduction in cell number, and a lack of later-born neurons [32]. Previous research has confirmed that activation of Sirt1 by RSV is involved in self-renewal, multipotency, and fate determination of NPCs [46, 47]. We previously demonstrated that RSV pretreatment reversed inhibition of Hes1 expression in the hippocampus induced by alcohol exposure through up-regulation of Sirt1 [17]. We also demonstrated that RSV pretreatment reversed an LPS-induced reduction of Sirt1 and Hes1 in the hippocampus, which may be involved in modulation of the division patterns of RGLs.

In contrast, we also found that RSV alone has no effect on RGL division mode or Hes1 expression. Inhibition of NF-κB signaling by Sirt1 deacetylase and its agonist, RSV, is strongly neuroprotective [48, 49]. Additionally, we observed that RSV pretreatment also inhibited the LPS-induced increase in NF-κB in the hippocampus. A previous study demonstrated that acute and chronic stress-induced activation of NF-κB signaling contributes to decreased proliferation of RGL progenitor cells [50]. We hypothesized that RSV-induced activation of Sirt1 contributes to the effects of NF-κB in reversing LPS-induced decreases in hippocampal neurogenesis. Recent evidence demonstrated an association between the Sirt1 gene and major depressive disorder [51]. Aberrant expression and/or function of Sirt1 may contribute to the pathophysiology of depression via neuroinflammation and dysregulation of neurogenesis. Further research is needed, in order to clarify the mechanisms of Sirt1 mediated RGL protection and modulation of division patterns.

In conclusion, our data provide the first evidence that RSV-induced activation of Sirt1 reverses LPS-induced depression-like behaviors by enhancing neurogenesis. RSV activation of Sirt1 has unique roles in promoting proliferation and increasing the available RGL progenitor cells in the GCL by stimulating symmetric division. Our findings also provide a new model for understanding the promising role of RSV in treating depression by promoting neurogenesis.

## MATERIALS AND METHODS

### Animals

Adult male C57/BL6 mice were provided by the Third Military Medical University and were housed grouped in a temperature-controlled room with a standard 12-h light/12-h dark cycle and *ad libitum* access to food and water. Eight-week-old mice were used at the start of the experiments. All experimental procedures were approved by Third Military Medical University and were performed according to the guidelines of laboratory animal care and use. All efforts were made to reduce the number of animals used and to minimize their discomfort.

### Drug treatment

RSV (Sigma-Aldrich, R5010, St. Louis, MO, USA) was dissolved in DMSO (Sigma-Aldrich) and ethanol 50% V/V and diluted in saline at a concentration of 20 mg/mL [52]. LPS from *Escherichia coli* 026:B6 (Sigma-Aldrich) was dissolved in 0.9% NaCl at a concentration of 1 mg/mL. Mice were randomly divided into the following 4 groups: (1) pretreatment with DMSO, followed by normal saline (0.9 % NaCl) (Saline+DMSO), (2) pretreatment with RSV, followed by normal saline (0.9 % NaCl) (Saline+RSV), (3) pretreatment with DMSO, followed by LPS (LPS+DMSO), or (4) pretreatment with RSV followed by LPS (LPS+RSV). RSV was administered by intraperitoneal (i.p.) injections (20 mg/kg) for 14 consecutive days, as previously described [52]. The control groups received an equivalent injection of DMSO. After 14 consecutive days of RSV or DMSO administration, mice also received i.p. injections of saline or LPS (1 mg/kg) for 5 consecutive days. The dose of LPS was based on a previous study [53].

Bromodeoxyuridine (BrdU; Sigma-Aldrich) was dissolved in 0.9% NaCl [17]. In order to assess cell proliferation in the DG, mice received BrdU every 2 hours, for a total of 3 injections at a dose of 100 mg/kg, and were then sacrificed 24 h after the first BrdU injection. In order to analyze neurogenesis in the DG, mice were sacrificed 2 weeks after the first BrdU injection, and BrdU^+^ cells as well as BrdU^+^ cells colabeled with doublecortin (DCX) were quantified.

### Behavioral experiments

All the behavioural tests began 12 h after the last LPS injection. Mice were acclimatized to the experimental room for at least 30 min prior to each test. Besides, the less stressful test (open field test) was conducted prior to the more stressful tests (elevated plus maze, forced swim test, tail suspension test).

#### Forced swimming test (FST)

Twelve hours after the last LPS or saline administration, mice completed the forced swimming test (FST) according to previous methods, with minor modification [54]. Briefly, each mouse was placed individually in a cylinder (height: 20 cm, diameter: 10 cm) for 6 min, which was filled with 10 cm height of water that was 25 °C in order to avoid a temperature-related stress response. Immobility durations were determined by recording the time that the mice remained floating in the water in an upright position and stopped struggling, typically moving slowly to keep their heads above water. The immobility durations were recorded and analyzed during the last 4 min of the 6 min test. Increased duration of immobility indicated a state of helplessness.

#### Tail suspension test (TST)

The tail suspension test was performed according to previous methods, with minor modification [55]. In brief, mice were suspended by a hook that was both acoustically and visually isolated, and which was 50 cm above the floor. The hook was placed approximately 1 cm from the tip of the tail. Mice were suspended for 6 min, and the immobility duration was recorded and analyzed during the last 4 min of the 6 min test.

#### Open-field test (OFT)

The open-field test was performed according to previous methods [56], using an open-field activity system (Biowill, Shanghai, China) and activity software (Biowill). Mice were placed in the center of the open-field box and activity was recorded for a period of 5 min. The total and center-area distances were measured and the duration of time in the central area was recorded. After each test, a solution of 10% alcohol was used to clean the square arena.

#### Elevated plus maze

The elevated plus maze was used to assess anxiety-like behavior. According to a previous protocol [57], the mouse was initially placed in the center area facing an open arm. The duration of time for the mouse to enter any of the 4 arms was recorded when all 4 paws crossed from the central region into an arm. The duration of time spent in the open arms and the number of total arm entries during the 5 min testing period were recorded.

### Immunohistochemistry and immuno-fluorescence

According to our previous methods [36], mice were deeply anesthetized with an overdose of isoflurane and transcardially perfused with 0.01 M phosphate-buffered saline (PBS, pH 7.4) for 5-10 min, followed by 4% paraformaldehyde in 0.1 M phosphate buffer (PBS, pH 7.4) for 15-20 min. Whole brains were removed and post-fixed in the same fixative for 3-4 d at 4 °C followed by 30% sucrose treatment at 4 °C. Serial coronal brain sections (25 μm in thickness) were cut on a cryostat and stored at −20 °C in cryoprotectant solution (30% ethylene glycol, 30% sucrose in 0.01 M PBS). The sections were first washed in 0.01 M PBS to remove the cryoprotectant solution, and then incubated in 3% H_2_O_2_ for 30 min at room temperature to quench endogenous peroxidase, followed by washing with 0.01 M PBS. The sections were then incubated with primary antibodies including rabbit anti-Iba1 (1:1000, Wako, CA, USA) or goat anti-DCX (1:200, Santa Cruz Biotechnology, CA, USA), in 1% bovine serum albumin (BSA) and 0.1 % Triton X-100 (12 h, 4 °C). BSA (1 %) replaced the primary antibodies in the negative controls. After washing, the sections were incubated with biotinylated secondary antibody (1:200, Dako, Glostrup, Denmark) (2 h, 37 °C), followed by the avidin- biotin complex (Dako). Finally, staining was visualized and photographed under a Zeiss Axiovert microscope (Oberkochen, Germany) equipped with a Zeiss AxioCam digital color camera connected to the Zeiss AxioVision 3.0 system.

For BrdU and GFAP or BrdU and DCX double staining, the sections were treated with 2 N HCl at 37 °C for 30 min to denature the DNA, followed by washing with 0.01 M PBS 3 times for 10 min before being incubated with mouse anti-BrdU (1:200, BD Biosciences, San Jose, CA, USA) and rabbit anti-GFAP (1:200, Zhongshan, Beijing, China) or with mouse anti-BrdU (1:200, BD Biosciences) and goat anti-DCX (1:200, Santa Cruz Biotechnology) in 1% BSA and 0.1% Triton X-100 for 12 h at 4 °C. After washing, sections were incubated with fluorescence-tagged secondary antibodies including Cy3 (1:500, donkey anti-mouse, Jackson ImmunoResearch, West Grove, PA, USA) and Alexa 488 (1:500, donkey anti-rabbit, Jackson ImmunoResearch) or Cy3 (1:500, donkey anti-goat, Jackson ImmunoResearch) and Alexa 488 (1:500, donkey anti-mouse, Jackson ImmunoResearch) for 2 h at 37 °C. Nuclei were subsequently stained with 4′,6-diamidino-2-phenylindole (DAPI, Beyotime, China). For SOX2 and GFAP double staining, sections were washed 3 times with 0.01 M PBS for 10 min before being incubated with rabbit anti-SOX2 (1:1000, Abcam, UK) and mouse anti-GFAP (1:500, Millipore, Temecula, CA, USA) in 1% BSA and 0.1% Triton X-100 for 12 h at 4 °C. Sections were then incubated with the fluorescence-tagged secondary antibodies Alexa 488 (1:500, donkey anti-mouse, Life Technologies, USA) and Cy3 (1:500, donkey anti-rabbit, Jackson ImmunoResearch) for 2 h at 37 °C). Nuclei were subsequently stained with DAPI. Sections were visualized using a confocal laser-scanning microscope (Leica TCS-SP2, laser lines at 488, 543, 633, Heidelberg, Germany) and analyzed with Leica imaging software.

### Western blotting

Hippocampus samples were isolated and homogenized in ice-cold RIPA lysis buffer (Beyotime, Shanghai, China). After centrifugation of lysates (15,000 *g*, for 5 min at 4 °C), the protein concentration was determined using a bicinchoninic acid kit (Beyotime Institute of Biotechnology, Shanghai, China). Protein samples (30 μg per lane) were separated on a 12% SDS-polyacrylamide gel at 80 V for 120 min, and then transferred onto polyvinylidene fluoride (PVDF) membranes at 250 mA for 60 min. The membranes were blocked with Tris-buffered saline (TBS) containing 0.1% Tween 20 (TBST) and 5% fat-free milk for 3 h at room temperature. The membranes were then incubated (overnight at 4 °C) with rabbit antibodies against Hes1 (1:1000, Chemicon, CA, USA), Sirt1 (1:1000, Chemicon), and NF-κB (1:2000, BD Biosciences), as well as a mouse antibody against β-actin (1:2000, Cell CWBIO, Beijing, China). Membranes were then incubated for 1 h at room temperature with a peroxidase-conjugated goat anti-rabbit immunoglobulin G (IgG; 1:2000, Santa Cruz Biotechnology) or goat anti-mouse immunoglobulin G (IgG; 1:2000, Santa Cruz Biotechnology). All western blotting data are representative of at least 3 independent experiments. Specific protein bands on the membranes were visualized by the enhanced chemiluminescence (ECL) method (Amersham, Piscataway, NJ, USA), according to the manufacturer's instructions. The relative intensities of Hes1, Sirt1, and NF-κB were normalized to the internal reference protein β-actin. Three animals per group were used for analysis.

### Cell counting and unbiased stereology

According to our previous methods [36], an average of five sections per mouse was analyzed to measure the number of Iba1^+^, DCX^+^, SOX2^+^/GFAP^+^ and SOX2^+^/GFAP^-^ cells in the SGZ (at the junction between the granule cell layer and the hilus). Five animals per group were used for analysis.

According to the methods of Roughton et al. [58], stereological cell counting was performed for quantification of the total number of BrdU^+^ cells in the SGZ, BrdU^+^/GFAP^+^ and BrdU^+^/DCX^+^ cells in the GCL (plus SGZ). Serial 25 μm sections through the rostrocaudal extent of the DG were selected at ten-section intervals for immunofluorescent staining and counterstaining with DAPI to mark nuclei in the DG. The total sum of the BrdU^+^, BrdU^+^/GFAP^+^ or BrdU^+^/DCX^+^ positive cells traced were multiplied by positive cells in the SGZ or GCL (plus SGZ) per section and series number to give the total number of BrdU^+^, BrdU^+^/GFAP^+^ or BrdU^+^/DCX^+^ positive cells in the DG. Five animals per group were used for analysis.

### Cell alignment analysis

We identified RGL progenitor cells as GFAP-positive cells that extend a single process from the SGZ toward the molecular layer. Cell alignment of BrdU-labeled RGLs was analyzed in order to evaluate the mode of cell division. Briefly, the vertical (asymmetrical) division mode is defined as daughter cells that are aligned vertically to the axis of the radial glial fiber, while horizontal (symmetrical) division mode describes daughter cells that are aligned horizontally to the axis of the radial glial fiber.

### Statistical analyses

Statistical analyses were performed using SPSS 17.0 software (SPSS Inc., Chicago, IL, USA) to conduct two-way ANOVAs. Significant effects were evaluated with Tukey's post hoc tests or Bonferroni corrections. Data are presented as mean ± SEM. Statistically significance was set at *p* < 0.05.
